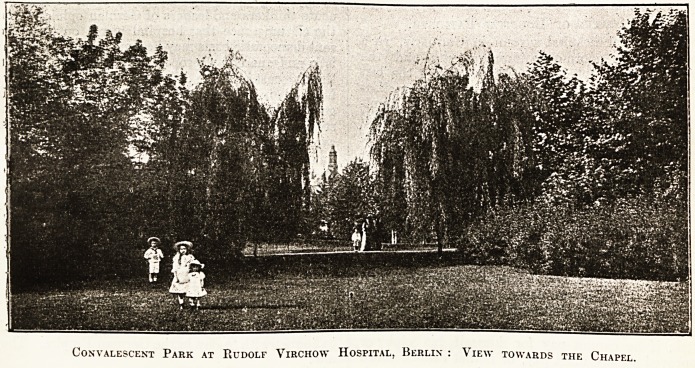# Government (Teaching) and Municipal (Non-Teaching) Hospitals


**Published:** 1912-10-19

**Authors:** Henry Burdett


### GOVERNMENT (Teaching) and MUNICIPAL (Non-Teaching) HOSPITALS.*

By SIR HENRY BURDETT, K.C.B.

### II. The Present Position of Hospitals in Germany.

In order to understand the position of hospitals in Germany to-day it is essential to examine a little closely into the effect which social insurance legislation has had upon the development of the hospital system. Twenty-five years ago the German hospitals, which were available for the general public, roughly and for the most part were shunned, being regarded mainly in the character of pauper asylums. There were, at this date, military, naval, and polyclinic hospitals and institutions, officered and managed and wholly or chiefly supported by the State, the funds being provided by the War Office, by the Empire, or by the provincial war chests. Further than this, there were private hospitals belonging to charitable corporations, religious bodies, trusts, associations, or societies, supported wholly or in part by voluntary effort, though they might be, and often were, subsidised by the municipality. Their management was in the hands of elective bodies chosen by the subscribers or others interested in the institution, and they were for the most part religious institutions or endowed hospitals. In addition to these there were private clinics belonging to private individuals, associations, or companies, run as business undertakings, for which in some cases, where financial success was not otherwise obtainable, support was sought from voluntary sources. The military and naval hospital accommodation was not available for the public, and although the public might obtain admission to a private clinic, the charges at most of these clinics were prohibitory, so far as the large majority of people were concerned. No self-respecting member of the working or middle classes would enter one of the general hospitals, which, as we have said, were regarded by them as pauper asylums or homes for the dying. The prejudice was also great against the charitable hospitals or religious houses, the management of which was not generally approved, and admission to which was regarded as the acceptance of charity, an indignity which no member of the German artisan or middle classes would suffer. At the time we refer to the management of most hospitals left much to be desired, and in matters of construction, hygiene, and general administration they did not approach the standard of the better type of hospitals to be met with in other countries. Such was the position of public opinion and of hospital accommodation in Germany a quarter of a century ago, at the time when insurance legislation was first introduced into that country. It is interesting to make this point perfectly clear because the passing of the National Insurance Act last year has created a great deal of discussion throughout the United Kingdom in regard to the future of the hospitals in this country. Indeed, the changes which may be expected to result in hospital provision, as a direct consequence of such legislation, cannot be few or unimportant.

CONVALESCENT PARK AT RUDOLF VIRCHOW HOSPITAL, BERLIN: VIEW TOWARDS THE CHAPEL.

### SOME EFFECTS OF NATIONAL INSURANCE.

In studying the effects of National Insurance in Germany and its probable effects in Great Britain it is essential to realise the main distinction between

* The first article of this series appeared in our issue of October 12, 1912.

the German and the English hospitals, which is to be found in the relative positions which they occupy in the public estimation. In England we are constantly endeavouring to explain to the public the necessity of hospitals, but in Germany, after twenty-five years of social insurance legislation, it has become an axiom, admitted as readily by the public as the corresponding axiom that isolation is necessary in cases of infectious disease, that hospitals are imperatively required, that they fulfil a useful object, and that they are worth supporting in every way. Hospital appeals in Germany are few and have become a diminishing quantity, showing, as we pointed out in our previous article, that the whole trend of opinion in Germany is in favour of the supersession of private venture institutions, which must either disappear altogether, or become standardised by shedding the abuses in management and otherwise which at present attach to many of them, that they, by co-operation and reorganisation, may one day form a part of the national system.

### The Status of Hospital Patients.

The present hospital system in Germany, as a result of the enormous improvements and the awakening of public opinion there during the last twenty-five years, has become a careful systematisation of co-operation between the State and municipality on the one hand, and the communal authorities and voluntary institutions on the other, to secure the best results for the sick within the German Empire. It is essential to speak of the sick, and not of the sick poor. "The German idea of a hospital is an institution where the best treatment is afforded to the citizen who is unwilling or unable to make his own terms with a private doctor at a cost which is well within the means of the middle classes and those who have been thrifty enough to provide for the days of sickness and disease. In the proper sense of the term there are no hospital poor in Germany; no patient is treated free of charge in a ward belonging to a public hospital; if he cannot pay for himself his club or approved society must pay, or if that cannot afford to do so, or if he is not a member of some such organisation, the communal authorities must pay his hospital bill. In theory the last-mentioned provision operates exactly in the same way as does the pauper provision in Great Britain. In practice it works quite differently. It is easy enough to see the immense advantage a hospital gains when it no longer bears the stigma of a pauper infirmary or charity. Equally important is the moral effect of such a *régime* of paying patients upon those who are admitted to the wards. In the lower classes of wards—which are nevertheless paying wards in the strict sense of the term—the patients cannot differentiate between themselves; they cannot point to Number So-and-So as a pauper patient who is being paid for by the ratepayers. Only the officials know the position and social standing of the patients, and in the eyes of their fellow-patients they are all equal and all enjoy the same privileges and the same comforts." This equality of status among the hospital patients in German hospitals in the lower-class wards is secured and maintained by the circumstance that they are all supplied on admission, whatever their outside status may be, with an outfit of clothing for day and night use, which is identical in every particular. The quality of this clothing in the best-managed German hospitals is of the best. The enforcement of the use of baths, ablutions, and personal cleanliness on the part of all the patients, both on admission and during their residence in the hospitals, is a material factor in this connection. This is so, for even the "Weary Willies" of the outside world, when they become hospital patients, are speedily converted so far as appearance is concerned into respectable, and it may be even into attractive, members of the hospital community. So complete is the equality thus established and maintained between the in-patients that, with the multiplication of hospitals of the highest type, which is one of the most remarkable features of social life in the Germany of to-day, a possible danger may arise, it is feared by some of the most acute thinkers and leaders of German opinion, from the circumstance that hospital wards on their present democratic basis may become centres for socialistic and anarchical propaganda. Force is given to this forecast by "the difference of standing between hospitals in this country and those in Germany. In general the German institution is a more sedate and steady place; it does not hurry itself unduly; the average length of stay of its patients is longer than in English hospitals of the same standing and size; its methods are in some ways uneconomical, not to say wasteful; and it provides for the immediate present, and does not pay attention to the future. The day-room in a German hospital is relatively large; the convalescent home, if it exists, in connection with the institution usually ridiculously small for the number of beds at the parent hospital."

### Various Types of German Hospitals.

We will next explain the five types of hospitals to be met with to-day in the German Empire. They are:

1. Military and naval hospitals.
2. Hospitals with polyclinics and medical schools attached which receive subsidies from the Treasury or Government. Amongst this type of hospital are numbered the most important centres of medical education, including universities and medical schools of world-wide repute.
3. Public hospitals or institutions which are subsidised by the municipal or provincial State authorities and are controlled by boards of management appointed by those authorities. Apart from patients' payments—an important source of revenue—they receive their support almost entirely from the municipal treasuries.
4. *Private hospitals—that is, charitable corporations, religious bodies, trusts, associations or societies supported wholly or in part by voluntary effort, though they may be and often are subsidised by the municipality.
5. *Private clinics run mainly as business undertakings for profit, the nature of which has been already indicated. This class includes all nursing and maternity homes.

[*N.B.—In German hospital statistics the institutions of the fourth and fifth type are lumped together under the heading of "private hospitals of more than eleven beds," a fact which it is essential to remember in dealing with the German reports and statistics in this connection.]

We have already indicated sufficiently the nature of types 1, 2, 4, and 5. As to type 3, which is by far the most numerous, and which includes the finest and best examples of hospital development in Germany, it may be well to enter into fuller details. The illustration which accompanies the present article gives an indication of the results achieved by the gardeners of the hospitals of Berlin. The Rudolf Virchow Hospital is not situated in the suburbs; it is a town hospital under the council of magistrates, and yet the grounds and gardens in the season, and indeed always, are attractive and hope-inspiring. The hospital has the merit or demerit of many of this type of hospital, that it is very large and contains some 1,700 beds. We shall have something to say in regard to size in subsequent articles. British readers who are interested in hospitals will be familiar with the Eppendorf Hospital at Hamburg, another hospital of this type, which has been written about in the English papers, and was, when it was built, in advance of most hospitals of its class. That hospital contains upwards of 2,200 beds, and our observation goes to prove that it is now superseded as the standard hospital of its type by, at any rate, the Munich Schwabing Hospital, of which the greater portion has been built, though the actual number of beds in occupation at present does not much exceed 800. Schwabing is a hospital which everyone who really wants to see the German hospital at its best should make a point of visiting. Visitors will there find many things to attract and, no matter what their experience of hospitals may be, something to learn.

### An Instructive Contrast.

The contrast between a hospital like Schwabing and one of the older Stadt hospitals against which the working classes had so just a prejudice, as already mentioned, was strikingly brought home to the writer during his recent visit to Berlin. There, in one of the older of the hospitals, where the nursing is supposed to be under a system producing the best results, he visited a large pavilion and found the wards overcrowded, and that the patients had been allowed to overflow and to overcrowd the day-room. Here the atmosphere and the whole surroundings and circumstances of the patients brought back vividly to the visitor's mind the condition and atmosphere of the sick wards in many a workhouse of the old type. Not for a quarter of a century had we seen or experienced anything quite so disheartening and disagreeable as the actual condition of these particular wards in fact was. The excuse which might be made, and probably would be urged, no doubt, is, that the older people who have to be provided for by the municipality must be put somewhere. That plea is justifiable, no doubt, but it will not excuse or explain the overcrowding of septic cases in the way in which we experienced it, nor will it justify the wrong and highly improper use of the day-room space for the reception of cases of this kind at all. Nor will it justify the admission of patients in numbers in excess of the accommodation permissible in a ward or within the space contained in the day-room of the average pavilion of a municipal hospital in Germany. We have mentioned this latter fact by way of contrast, and indirectly by way of explanation of the just prejudice which prevailed twenty-five years ago amongst the working classes throughout Germany against public hospitals subsidised by the municipal or provincial State authorities.

(*To be continued.*)

## Figures and Tables

**Figure f1:**